# Genetic diversity of cattle in three regions of Gauteng province in South Africa using microsatellite markers

**DOI:** 10.1007/s11250-026-05092-9

**Published:** 2026-06-15

**Authors:** P. Soma, B. B. Kooverjee, A. Yika

**Affiliations:** 1 Agricultural Research Council – Animal Production, Department of Animal Breeding and Genetics, Pretoria, South Africa; 2 Department of Animal Sciences, University of the Free State, Bloemfontein, South Africa

**Keywords:** Genetic diversity, Genetic distance, Microsatellite markers, Non-descript cattle, Population structure

## Abstract

Livestock production in South Africa remains an important contributor to ensuring food security and provides many social and economic attributes to the country. Locally adapted and indigenous breeds are able to survive in extreme weather conditions and produce and reproduce for long periods of time. Cattle (non-descript) in these areas constitute a valuable genetic resource. The study was conducted to assess the genetic diversity and population structure of cattle populations in three regions of Gauteng. Hair samples were collected from Western Gauteng (*n* = 266), Lesedi (*n* = 132) and Tshwane (*n* = 69) regions. Direct polymerase chain reaction (PCR) was conducted using eleven International Society for Animal Genetics (ISAG) recommended bovine microsatellite markers. Fragment analysis was performed. Five local cattle breeds (*n* = 473) were included as reference populations (Afrikaner, Nguni, Brahman, Bonsmara and Drakensberger breeds). The Angus breed was included as an outgroup. Statistical analysis was performed using MS Toolkit, STRUCTURE and GenAlEx programs. High level of genetic diversity was found across the populations, with an average expected heterozygosity of 75%. Levels of inbreeding varied from − 0.031 to 0.042 with a mean of 0.015, illustrating low levels of inbreeding within the populations. The results from this study indicated that more genetic differentiation found within populations rather than between populations. These findings may serve as a baseline for the management of genetic resources and contribute in developing future breeding programmes in these municipalities.

## Introduction

South Africa (SA) is richly endowed with a number of cattle breeds. Indigenous cattle breeds play a significant role in the livelihoods of smallholder farmers across the country and Gauteng province. The livestock sector in Gauteng contributed 75.5% to the gross farming income in the commercial agriculture industry in 2017 (Statistics South and Africa 2020). This highlights the importance of livestock in Gauteng province and making it an essential resource for emerging and small scale farmers. Many farmers located in the various municipalities within Gauteng, practice cattle farming as a source of income and means of sustaining livelihoods. These herds comprise of various indigenous cattle breeds and their crosses, namely Nguni and Bonsmara. Indigenous cattle require less input from the farmer and are able to survive on limited water and low feed quality (Sanarana et al. 2016). Nguni cattle are known for their heat tolerance, parasite resistance and ability to walk long distances in search of food and water (Tada et al. 2013). The Bonsmara cattle are known for high fertility, pathogen resistance and well adapted to South Africa’s climatic conditions (Corbet et al. 2006; Van Marle-Köster et al. 2021). Mixed farming systems may lead to the loss of genetic diversity within a population, resulting in highly inbred populations (Santana et al., 2014). An increase in the level of inbreeding may negatively affect the reproductive performance and growth of animals, and in the long term, result in decreased milk and beef production (Reverter et al. 2017).

The conservation of livestock diversity is essential to meet future needs in Africa and Southern Africa. To be able to manage an unpredictable future, genetic reserves capable of readily responding to directional forces imposed by a broad spectrum of environments must be maintained. Maintaining genetic diversity serves as an insurance package against current adverse conditions and climate change. Due to diversity among different environments, nutritional demands and challenges from infectious agents, a variety of breeds and populations are required (Kabi et al. 2016). These act as storehouses of genetic variation forming the basis for selection in times of biological stress such as drought or disease epidemics (Mwai et al. 2015). Several studies have focused on the genetic characterisation of commercial breeds (Sanarana et al. 2016; Pienaar et al. 2018). Limited studies have reported the genetic status of cattle in rural areas of South Africa (Mamogobo et al. 2020), in particular Gauteng province. Thus, the aim of this study was to assess the genetic diversity and population structure of cattle populations in three regions of Gauteng using microsatellite markers.

## Materials and methods

### Study population and sample collection

**Fig. 1:**
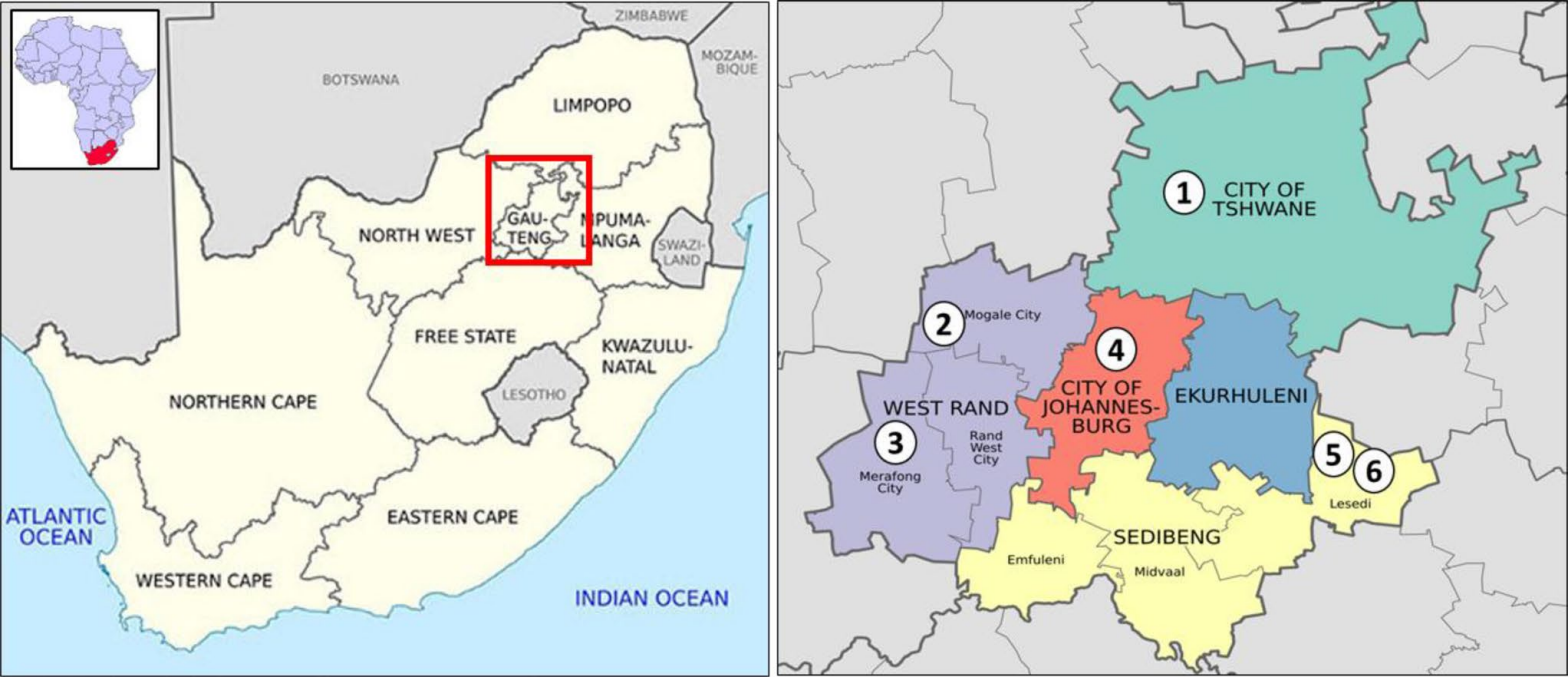
Geographical location of study populations, where each population is numbered from 1–6. The populations are as follows: (1) Tshwane, (2) Randfontein; (3) Merafong; (4) City of Johannesburg; (5) Lesedi A; and (6) Lesedi B

The study was conducted on cattle populations from three regions of Gauteng, namely; Lesedi, Tshwane and Western Gauteng. The climate for these areas are characterized by warm, rainy summers and dry, cold winters which create very specific selection pressures, so both breed choice and management tend to converge on animals that can handle heat stress, parasites, and seasonal feed constraints. The specific elevation causes some variation across these regions. Tshwane is usually warmer at an altitude around 1300 m. Western Gauteng has cooler conditions at an altitude of 1700 m and Lesedi experiences cooler conditions in Summer. These regions were further divided into 6 populations based on geographical locations (Fig. 1).

[Image adapted from http://www.southafrica-canada.ca/south-africas-nine-provinces/ and https://en.wikipedia.org/wiki/List_of_municipalities_in_Gauteng#/media/File:Map_of_Gauteng_with_municipalities_named_and_districts_shaded_(2016).svg, accessed on 04/02/2024]

**Fig. 2:**
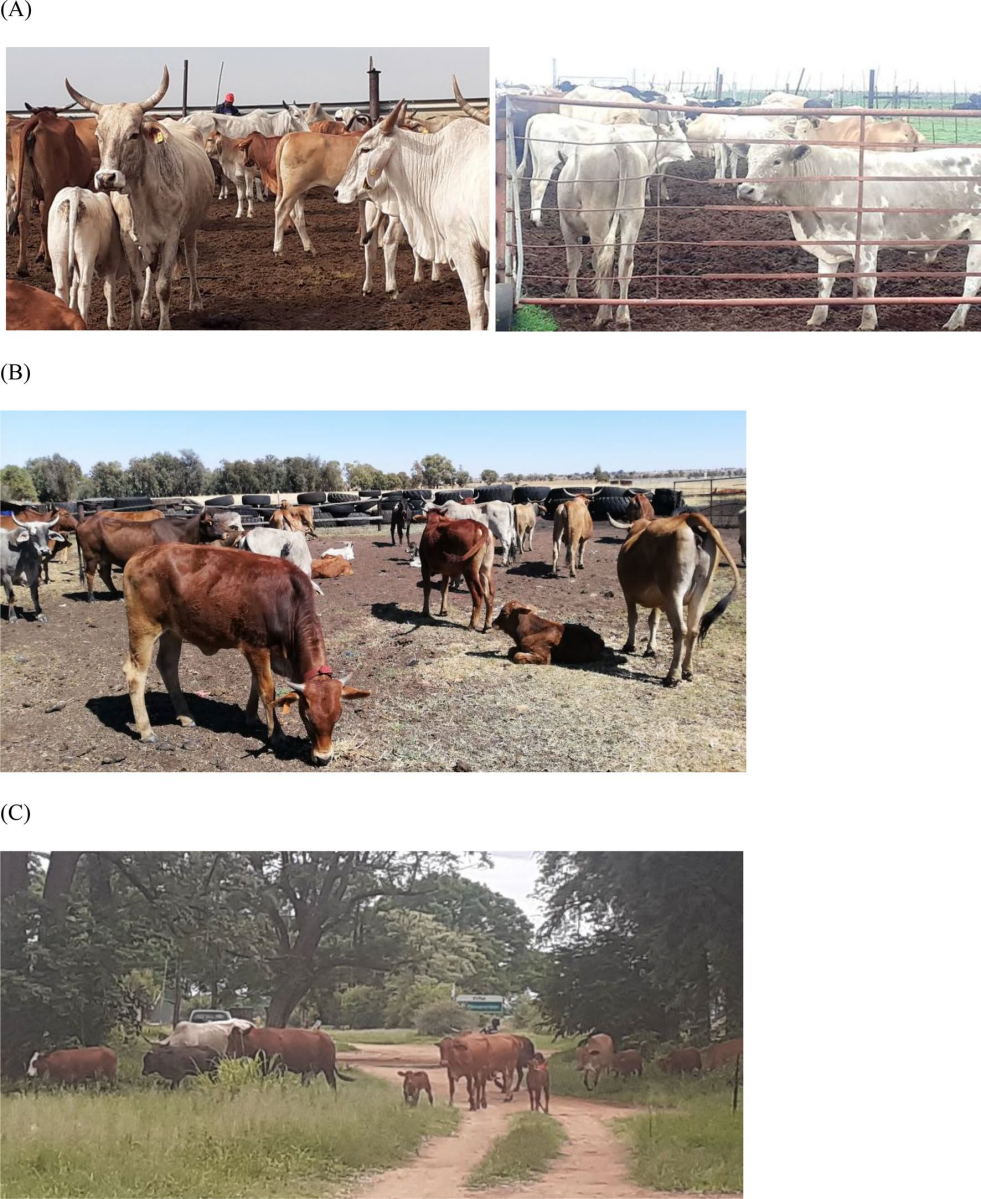
Photos of cattle from the (**A**) Western Gauteng, (**B**) Tshwane and (**C**) Lesedi areas

The Lesedi population comprised of three farms, where Lesedi A comprised of farms located in the Nigel area while Lesedi B included farms from the Heidelberg area. A total of 467 animals were selected (Lesedi A (LesA) *n* = 61, Lesedi B (LesB) *n* = 71, Tshwane (Tsh) *n* = 69, Randfontein (Ran) *n* = 71, Merafong (Mer) *n* = 87 and City of Johannesburg (COJ) *n* = 108) (Fig. 2). Hair samples were collected from the switch of the tail of the animal and was prepared for fragment analysis using a panel of 11 International Society for Animal Genetics (ISAG 2024) (www.isag.us (accessed on 20 May 2024) recommended microsatellite markers (Table 2).

**Fig. 3:**
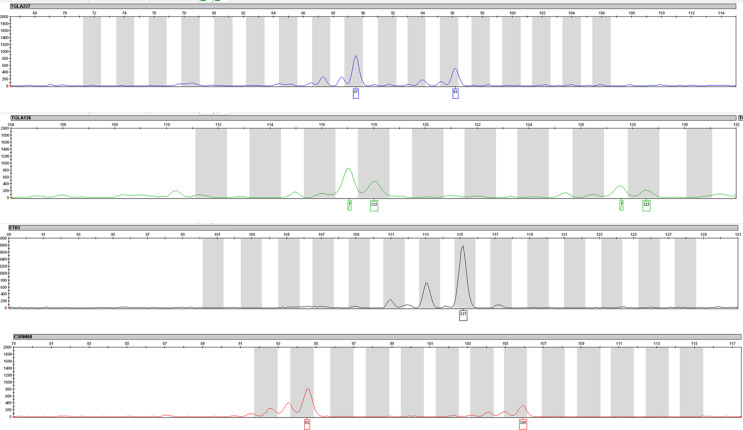
Electropherograms showing DNA profiles at four loci

### Microsatellite genotyping and analysis

Direct PCR was performed by cutting 6–8 hair roots per sample (Mohammed-Geba 2017). DNA amplification was performed using Gene Amp PCR System 9700 thermocycler consisting of the following reaction conditions: 10 min polymerase activation step at 95 °C, 33 cycles of 45 s denaturation at 94 °C, 90 s annealing at 61 °C, 60 s elongation at 72 °C and a final elongation step at 72 °C for 60 min. Fragment analysis was done on an ABI PRISM 3130xl Genetic Analyzer (Applied Biosystems, USA) to separate the amplified DNA fragments as per the manufacturer’s protocol. The resulting genotypes were scored using GeneMapper^®^ software version 5.0 (Applied Biosystems) (Fig. 3).

### Genetic diversity

The data was further interrogated using various population genetic programs. Mean number of alleles (MNA), private alleles (PA), observed heterozygosity (Ho), expected heterozygosity (He), polymorphic information content (PIC) and estimating Wright’s F-statistics (F_IS_, F_IT_, and F_ST_) for each marker were calculated using GenAlEx ver. 6.5 software (Smouse and Peakall 2012) and Excel Microsatellite (MS) Toolkit ver. 3.1 software (Park 2001). The latter software was used to verify the data for errors, duplication or genetically identical samples, and to generate input files for STRUCTURE software. The genetic distances between study populations and against reference breeds were estimated also using GenAlEx ver. 6.5 (Smouse and Peakall 2012). The genetics distances were plotted using the ‘ape’ v5.8 package (Paradis and Schliep 2019) in R v4.1.1 with the function of neighbor-joining tree estimation of Saitou and Nei (1987).

### Population genetic structure

Population structure analysis of study populations was assessed with principal coordinate analysis (PCA) using GenAlEx ver 6.5 (Smouse and Peakall 2012). Additionally, STRUCTURE ver. 2.3.4 (Pritchard 2009) based on a Bayesian clustering algorithm was utilized in determining the admixture present in each study population when compared to the reference breeds. Six SA cattle breeds, Afrikaner (*n* = 51), Nguni (*n* = 59), Brahman (*n* = 60), Bonsmara (*n* = 61), Drakensberger (*n* = 61) and Angus (*n* = 61) were included as reference populations. To determine a representative K value for the data, independent allele frequencies and an admixture model was used with a burn-in period of 50 000, followed by 100 000 MCMC iterations. Individual runs were done for each K value between 2 and 10, replicated 10 times. STRUCTURE HARVESTER was used to determine the most probable K value using DeltaK (ΔK) (Earl and Von Holdt 2012).

## Results

Eleven microsatellite markers were used to assess the genetic diversity of cattle from three regions in Gauteng. All targeted loci were successfully amplified with the correct PCR product sizes and were polymorphic in the six study cattle populations. A total number of 615 alleles were detected across the 11 microsatellite markers with an overall mean of 9.318 (Table 1). The number of alleles detected in the populations ranged from 95 (Tsh) to 105 (Mer). The locus with the highest number of alleles was TGLA53 while the lowest was BM1824.

**Table 1:** The selected ISAG microsatellite marker loci, chromosome location, allelic range and number of alleles per population

Locus	Chr	Allelic Range	Number of alleles per population					
		(bp)	LesA	LesB	Tsh	COJ	Ran	Mer
BM2113	2	124–146	11	11	10	10	11	11
ETH10	5	206–222	9	8	8	9	8	9
SPS115	15	247–261	6	8	6	9	8	7
TGLA227	18	76–104	10	9	10	8	11	9
TGLA53	16	151–187	13	16	14	16	16	14
INRA23	3	201–225	8	11	7	10	12	12
TGLA122	21	136–182	12	10	8	12	14	11
TGLA126	20	111–127	8	7	7	8	7	9
BM1824	1	176–188	8	7	7	6	7	5
ETH225	9	139–157	8	9	12	7	8	9
ETH3	19	100–128	7	7	6	6	9	9
Total			100	102	95	101	111	105

Chr: Chromosome location; LesA: Lesedi A; LesB: Lesedi B; Tsh: Tshwane; COJ: City of Johannesburg; Ran: Randfontein; Mer: Merafong

Descriptive statistics of genetic diversity within the six populations are summarized in Table 2. The mean number of alleles ranged from 8.63 ± 0.778 in the Tshwane population to 10.09 ± 0.899 in the Randfontein population. The expected heterozygosity’s ranged between 0.72 ± 0.03 (Lesedi A) and 0.79 ± 0.02 (Randfontein), while observed heterozygosity’s ranged from 0.71 ± 0.04 (Tshwane) to 0.75 ± 0.02 (Randfontein). Furthermore, low levels of inbreeding was observed across all populations ranging from − 0.03 to 0.04.

**Table 2:** Descriptive statistics of genetic diversity parameters in the cattle populations from Gauteng (Mean ± Standard Error (SE))

Populations	MNA ± SE	PA	H_e_ ± SE	H_o_ ± SE	F_is_ ± SE
Lesedi A	9.091 ± 0.653	0	0.722 ± 0.032	0.729 ± 0.040	-0.012 ± 0.035
Lesedi B	9.364 ± 0.801	0	0.757 ± 0.024	0.726 ± 0.033	0.041 ± 0.030
Tshwane	8.636 ± 0.778	2	0.732 ± 0.030	0.714 ± 0.040	0.027 ± 0.032
COJ	9.182 ± 0.872	1	0.748 ± 0.030	0.729 ± 0.025	0.023 ± 0.016
Merafong	9.545 ± 0.731	3	0.729 ± 0.029	0.750 ± 0.031	-0.031 ± 0.022
Randfontein	10.091 ± 0.899	0	0.790 ± 0.020	0.755 ± 0.022	0.042 ± 0.020
Mean	9.318 ± 0.789		0.746 ± 0.028	0.734 ±0.032	0.015 ± 0.026

MNA: Mean number of alleles; PA: Private alleles; H_e_: Expected heterozygosity; H_o_: Observed heterozygosity; F_is_: With-in population inbreeding estimate

The genetic relationship between the populations and reference breeds are shown in Table 3. The genetic distances between the studied populations indicated relatively close relationships (< 0.25), with the shortest distance between COJ and Randfontein (0.016), followed by COJ and Brahman (0.025) (Table 3). This finding suggested that these populations are more closely related. Among the study populations, Merafong and Tshwane were the most closely related (Fis = 0.027). The smallest overall genetic distance was between the Tshwane study population and the Nguni reference breed (Fis = 0.022). Overall, the low pairwise FSt values (< 0.05) among the six study populations indicated that the vast majority of genetic variation resided withine, rather than between, these groups.

**Table 3:** Pairwise estimates of genetic differentiation based on F_st_ among the reference and study populations

	Afr	Ang	Bon	Bra	Dra	LesA	LesB	Ngu	Tsh	COJ	Mer
Ang	0.197										
Bon	0.113	0.133									
Bra	0.155	0.170	0.146								
Dra	0.114	0.105	0.101	0.119							
LesA	0.065	0.109	0.031	0.106	0.064						
LesB	0.096	0.107	0.067	0.066	0.060	0.044					
Ngu	0.074	0.144	0.095	0.097	0.064	0.058	0.055				
Tsh	0.061	0.112	0.067	0.093	0.043	0.037	0.034	0.022			
COJ	0.102	0.118	0.094	0.025	0.071	0.058	0.038	0.054	0.049		
Mer	0.078	0.107	0.059	0.075	0.060	0.039	0.037	0.044	0.027	0.038	
Ran	0.077	0.097	0.064	0.043	0.043	0.033	0.026	0.036	0.026	0.016	0.024

Afr; Afrikaner; Ang: Angus; Bon: Bonsmara; Bra: Brahman; Dra: Drakensberger; Ngu: Nguni; LesA: Lesedi A; LesB: Lesedi B; Tsh: Tshwane; COJ: City of Johannesburg; Ran: Randfontein; Mer: Merafong

**Fig. 4:**
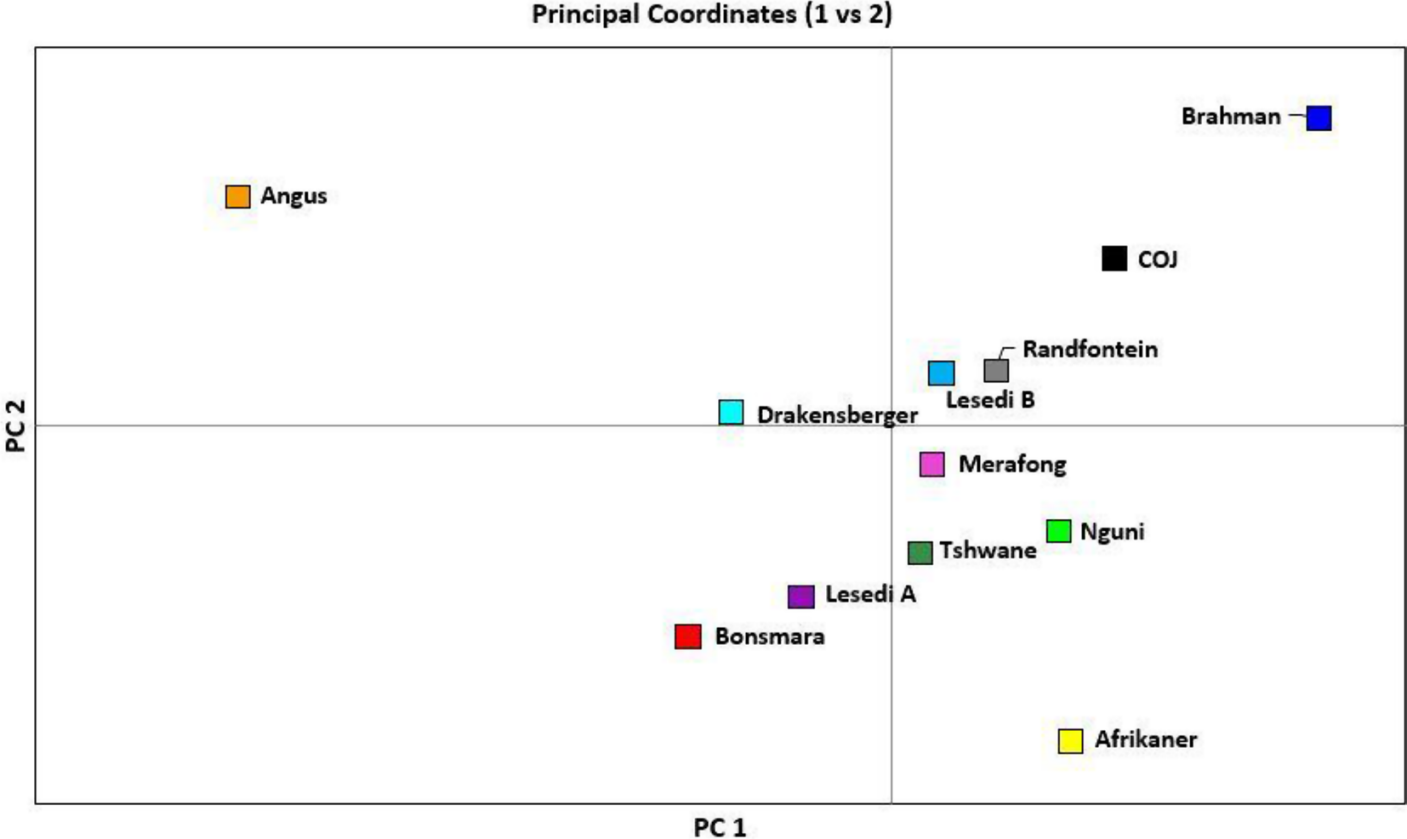
Principal Coordinate Analysis (PCA) via covariance matrix with data standardization for the cattle population in Gauteng

**Fig. 5a:**
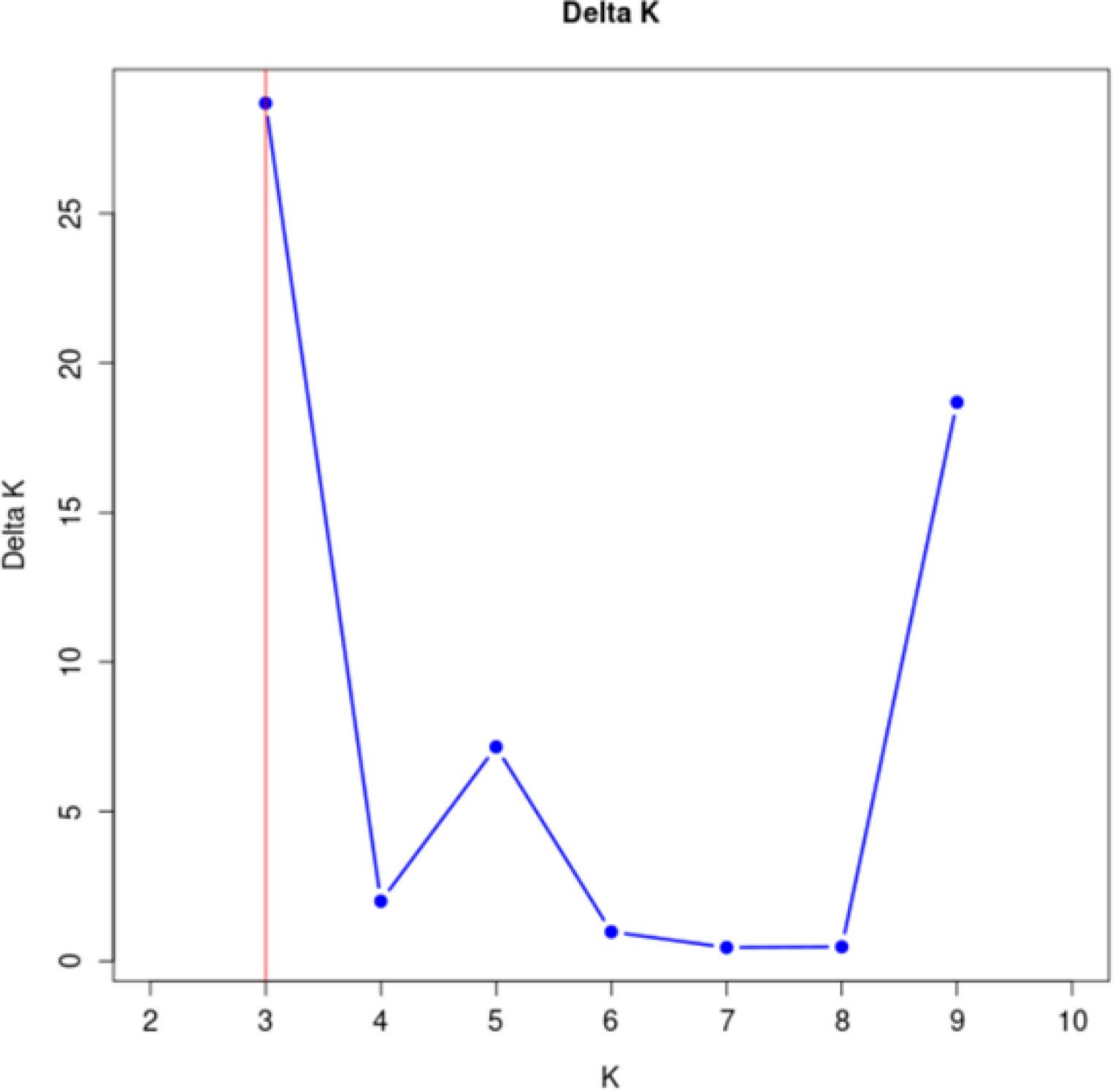
The Delta K value of each cluster was plotted to determine the optimum K value

**Fig. 5b:**
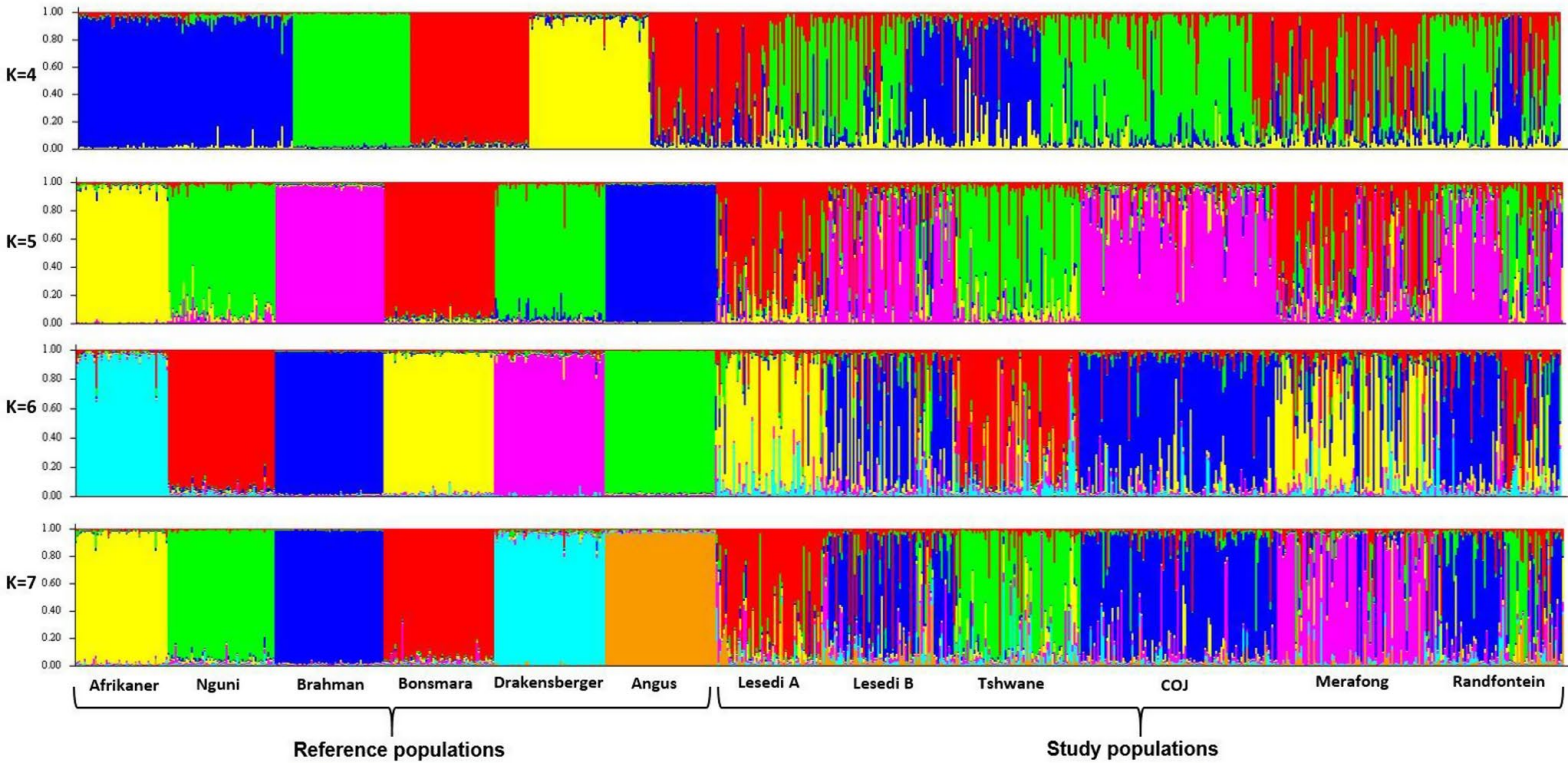
Estimated population structure of the cattle populations in Gauteng and six reference populations at *K = 4*, *K* = 5, *K* = 6 and *K* = 7, where *K* is the number of clusters present in the population

**Fig. 6:**
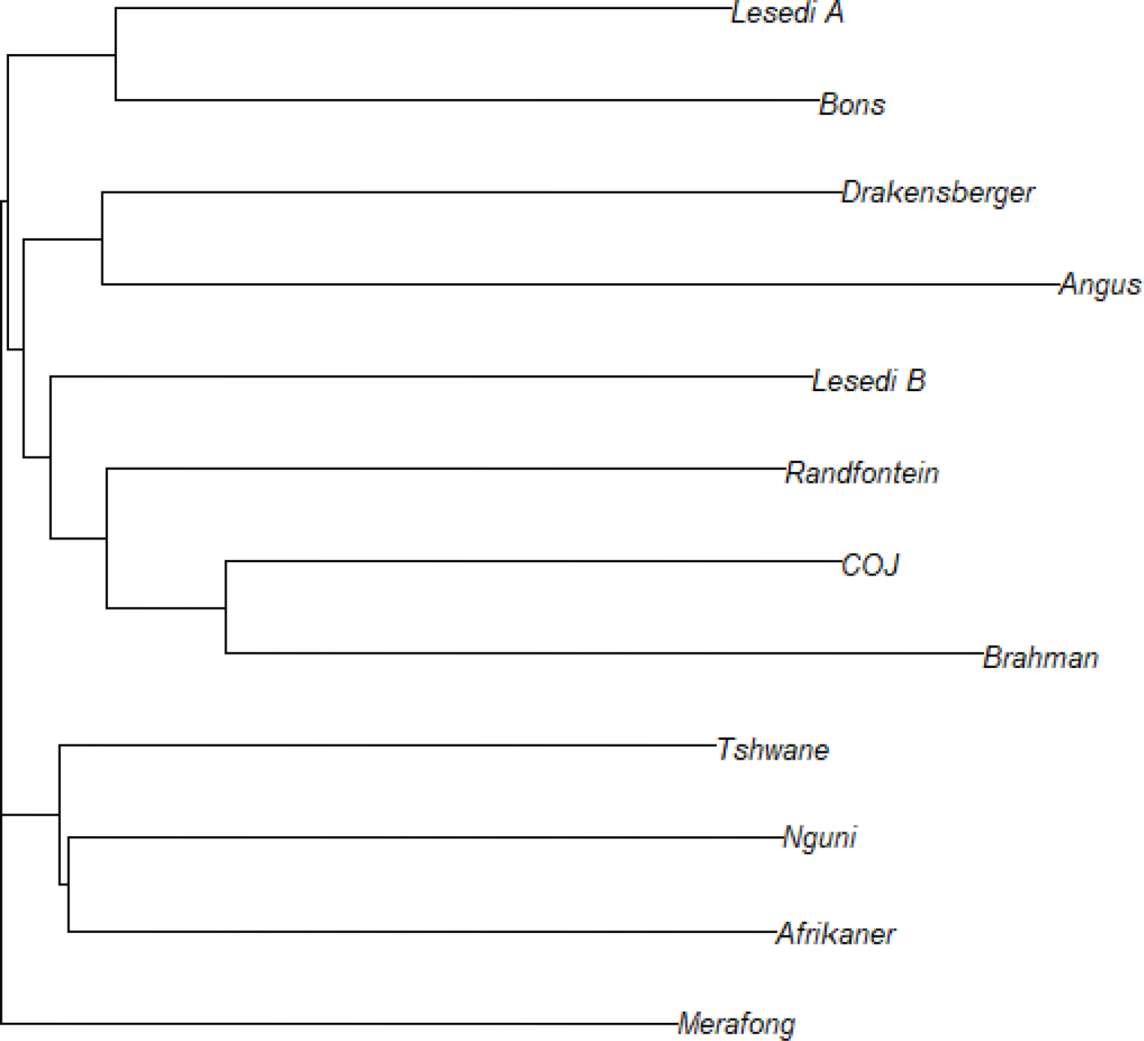
Phylogenetic tree depicting the evolutionary relationships among different breeds based on genetic distance. The tree was constructed using the Neighbor-Joining (NJ) method, with branch lengths representing genetic divergence. Closer branches indicate more genetically similar breeds, while longer branches signify greater genetic differentiation

The PCA plot (Fig. 4), shows the Tshwane population clustering in the same quadrant as the Nguni reference population and Merafong study population. The PCA plot also displays the Lesedi A clustering in the same quadrant as Bonsmara while Lesedi B, Randfontein and COJ clustering in the same quadrant as the Brahman reference breed. For estimation of the population structure and level of admixture, the estimated probabilities (Ln Pr) of the number of populations (K) were varied between 2 and 10. Based on the Ln Pr (X|K) value of K versus ΔK distribution (Evanno et al. 2005), the most probable number of populations are four and six (K = 6) (Fig. 5a). K = 4 revealed biologically meaningful subdivision. At K = 4, the clusters correspond to the geographic region and the replicate runs were consistent, suggesting that this level captures the relevant substructure beyond the primary partition.

The study populations and reference breeds clustered independently, with the former showing high levels of admixture. Furthermore, the population structure analysis shows that Lesedi A has more Bonsmara influence while Lesedi B has more Brahman influence, which coincides with the different breeding strategies applied at the respective farms (Figs. 5b and 6). Nguni influence was observed in the Tshwane population while Lesesdi B, COJ and Randfontein displayed Brahman influence. The Merafong population shows to have Bonsmara, Brahman and some Nguni influence at *K* = 6, however at *K* = 7 the Merafong population has a more admixed structure (Fig. 5b). This may be explained by the farmer’s choice of using a mixed breeding system, supporting the low inbreeding value present in the Merafong population. These results are further supported by the phylogenetic tree of all six populations as well as reference populations (Fig. 6).

## Discussion

Understanding the genetic diversity and relationships amongst cattle from various regions of Gauteng is important for successful animal production. The genetic diversity of a population is vital for its genetic improvement especially for adaptive traits and those linked with sustainable production when facing environmental disturbances such as warmer and dryer weather conditions (Nyamushamba et al. 2017). However, it is important to understand the current factors that influence population and diversity such as high levels of inbreeding, movement of cattle between herds, and sharing of bulls. In most rural areas of Gauteng, breeding remains uncontrolled and in the absence of pedigree records; and herds not being managed efficiently, may lead to the loss of high potential genetic resources. Hence, this study was focused on understanding the genomic structure of cattle populations in Gauteng.

An average observed heterozygosity of 73%, average expected heterozygosity of 75% and 9.31 alleles per locus was obtained across the study populations. The Ho and MNA in this study was higher than that reported by Mamogobo et al. (2020), which had Ho = 0.66 and MNA = 8.14. Expected heterozygosity and MNA was similar to Mamogobo et al. (2020), He = 0.75, MNA = 8.14), but higher than (Madilindi et al. 2020; He = 0.724, MNA = 7.2) and (Sanarana et al. 2016; He = 0.701, MNA = 6.47). The Randfontein population had the highest MNA indicative of high genetic diversity present in the population, while the lowest MNA was detected in the Tshwane population. Private alleles in this study defined as alleles unique to a population. Six private alleles were identified between Tshwane (2), COJ (1) and Merafong (3). Additionally, the inbreeding was low across the study populations with a mean of 0.015, which was lower than mean Fis = 0.018 reported by Van der Westhuizen et al. (2020). This could be a result of uncontrolled or mixed cross breeding strategies employed in these populations.

The PCA assessment clearly distinguished the study populations although showing a close relationship between them. The first principal coordinate separated Merafong from the other two western Gauteng populations (COJ and Randfontein) which clustered in the second principal coordinate. Furthermore, the two Lesedi populations clustered differently and were separated by the second principal coordinate – this is validated by the Structure plot which displays Bonsmara influence in Lesedi A and Brahman influence in Lesedi B. This observation is in line with the farmers preference of breeds used in their breeding practices. Angus (being of European ancestry) is the most distant population relative to the studied populations. However, Drakensberger was close to the study populations, which may be explained by the breed’s composition of 38% African taurine (Makina et al. 2016). The phylogenetic tree, further supported the clustering patterns seen in the PCA. For instance, the clade comprising of Tshwane study population, and the two reference population of Nguni and Afrikaner, which are Sanga type cattle. These Sanga cattle are well adapted to warmer climates, harsh environments and drought conditions (Chabalala 2024), making them better suited to areas like the Tshwane region. The clustering of COJ, Randfontein and Lesedi A close to the Brahman reference population, could indicate the use of Zebu cattle primarily for traits like cold-adaptation. Brahman cattle can adapt to cooler climates by growing a thicker coat to retain body heat (Scheffler 2022), unlike the other breeds, which makes them more suitable for the western Gauteng region.

The results of admixture in this study are consistent with the findings by Mamogobo et al. (2020) and Scholtz et al. (2008) that the majority (66.4%) of cattle herds that make up the communal and emerging sectors are non-descript or crossbred. Genetic resources of indigenous cattle breeds have been long held by traditional and communal farmers and have been the ultimate reason for the survival of the sector through challenges in extreme environmental factors (Nyamushamba et al. 2017). It is therefore cause for concern to observe a large extent of admixture in the study populations which may indicate a potential loss of essential genetic resources required for environmental adaptation and disease resistance. The admixture may be due to current breeding strategies employed by the farmers as well as the geographical location of the farms. Overall, the study populations showed great variability in terms of population structure.

In conclusion, this was the first study to conduct a genetic assessment of the cattle population in the three regions of Gauteng. Study populations revealed close genetic relation to all reference breeds, except Angus. However, Bonsmara and Brahman influence was detected in the Lesedi area, Nguni influence in Tshwane area and Brahman influence in the western Gauteng region. Overall, cattle in these regions of Gauteng are not purebred, but are instead mixed breed animals shaped by local breeding practices. The findings from this study may assist farmers in making sound decisions regarding the animals to be used in their cattle breeding programs. It is recommended that farmers develop region-specific breeding strategies that can build on existing breed influences that was found in this study to further improve herd performance and long-term sustainability. For example, use Brahman influence in western Gauteng to improve heat and tick resistance, or Nguni influence in Tshwane to enhance fertility and hardiness.

## Data Availability

The microsatellite datasets generated during and /or analysed during the current study are not publicly available due to ARC intellectual property practices, but are available from the corresponding author on reasonable request.

## References

[CR3] Chabalala NT (2024) The farm gate carbon and water footprint of diverse beef cattle genotypes in South Africa and its environmental impact (Master’s thesis, University of South Africa (South Africa))

[CR1] Corbet N.J., Shepherd R.K., Burrow H.M., Prayaga K.C., Van der Westhuizen J., Bosman D.J. (2006) Evaluation of Bonsmara and Belmont Red cattle breeds in South Africa. Genetic parameters for growth and fertility. Aust J Exp Agric 46:213–223. doi10.1071/EA05224

[CR2] Earl DA, Von Holdt BM (2012) STRUCTURE HARVESTER: a website and program for visualizing STRUCTURE output and implementing the Evanno method. Conserv Genet Resour 4:359–361. 10.1007/s12686-011-9548-7

[CR4] Evanno G, Regnaut S, Goudet J (2005) Detecting the number of clusters using the software STRUCTURE: a simulation study. Mol Ecol 14:2611–2620. 10.1111/j.1365-294X.2005.02553.x 15969739 10.1111/j.1365-294X.2005.02553.x

[CR5] International Society for Animal Genetics (2024) http://www.isag.us/ accessed on May 2024

[CR6] Kabi F, Muwanika V, Masembe C (2016) Indigenous cattle breeds and factors enhancing their variation, potential challenges of intensification and threats to genetic diversity in Uganda. Anim Genetic Resour 58:1–12. 10.1017/S2078633615000326

[CR7] Madilindi MA, Banga CB, Bhebhe E, Sanarana YP, Nxumalo KS, Taela MG, Magagula BS, Mapholi NO (2020) Genetic diversity and relationships among three Southern African Nguni cattle populations. Trop Anim Health Prod 52:753–762. 10.1007/s11250-019-02066-y 31529304 10.1007/s11250-019-02066-y

[CR8] Makina SO, Whitacre LK, Decker JE, Taylor JF, MacNeil MD, Scholtz MM, Van Marle-Köster E, Muchadeyi FC, Makgahlela ML, Maiwashe A (2016) Insight into the genetic composition of South African Sanga cattle using SNP data from cattle breeds worldwide. Genet Selection Evol 48:1–7. 10.1186/s12711-016-0266-1 10.1186/s12711-016-0266-1 PMC5111355 27846793

[CR9] Mamogobo MD, Mapholi NO, Nephawe KA, Nedambale TL, Mpofu TJ, Sanarana YP, Mtileni BJ (2020) Genetic characterisation of non-descript cattle populations in communal areas of South Africa. Anim Prod Sci 61:84–91. 10.1071/AN20030

[CR10] Mohammed-Geba K (2017) Direct PCR as a rapid and simple forensic technique for detection of DNA profiles from human hair samples. J Bioscience Appl Res 3:90–96

[CR11] Mwai O, Hanotte O, Kwon YJ, Cho S (2015) African indigenous cattle: unique genetic resources in a rapidly changing world. Asian-Australasian J Anim Sci 28:911. 10.5713/ajas.15.0002r 10.5713/ajas.15.0002R PMC4478499 26104394

[CR12] Nyamushamba GB, Mapiye C, Tada O, Halimani TE, Muchenje V (2017) Conservation of indigenous cattle genetic resources in Southern Africa’s smallholder areas: turning threats into opportunities—A review. Asian-Australasian J Anim Sci 30:603. 10.5713/ajas.16.0024 PMC5411820 27004814

[CR13] Paradis E, Schliep K (2019) ape 5.0: an environment for modern phylogenetics and evolutionary analyses in R. Bioinformatics 35:526–528. 10.1093/bioinformatics/bty633 30016406 10.1093/bioinformatics/bty633

[CR14] Park SDE (2001) The Excel microsatellite toolkit. From Trypanotolerance in West African Cattle and the Population Genetic Effects of Selection

[CR15] Pienaar L, Grobler JP, Scholtz MM, Swart H, Ehlers K, Marx M, MacNeil MD, Neser FWC (2018) Genetic diversity of Afrikaner cattle in southern Africa. Trop Anim Health Prod 50:399–404. 10.1007/s11250-017-1447-9 29043474 10.1007/s11250-017-1447-9

[CR16] Pritchard JK (2009) STRUCTURE ver. 2.3. http://pritch.bsd.uchicago.edu/

[CR17] Reverter A, Porto-Neto LR, Fortes MRS, Kasarapu P, De Cara MAR, Burrow HM, Lehnert SA (2017) Genomic inbreeding depression for climatic adaptation of tropical beef cattle. J Anim Sci 95:3809–3821. 10.2527/jas2017.1643 28992001 10.2527/jas2017.1643

[CR18] Saitou N, Nei M (1987) The neighbor-joining method: a new method for reconstructing phylogenetic trees. Mol Biol Evol 4:406–425 3447015 10.1093/oxfordjournals.molbev.a040454

[CR19] Sanarana Y, Visser C, Bosman L, Khathutshelo N, Maiwashe A, van Marle-Koster E (2016) Genetic diversity in South African Nguni cattle ecotypes based on microsatellite markers. Trop Anim Health Prod 48:379–385. 10.1007/s11250-015-0962-9 26611262 10.1007/s11250-015-0962-9

[CR20] Santana Jr ML, Pereira RJ, Bignardi AB, El Faro L, Tonhati H, Albuquerque LG (2014) History, structure, and genetic diversity of Brazilian Gir cattle. Livest Sci 163:26–33. 10.1016/j.livsci.2014.02.007

[CR21] Scheffler TL (2022) Connecting heat tolerance and tenderness in Bos indicus influenced cattle. Animals 12(3):220. 10.3390/ani12030220 35158544 10.3390/ani12030220 PMC8833572

[CR22] Scholtz MM, Bester J, Mamabolo JM, Ramsay KA (2008) Results of the national beef cattle survey undertaken in South Africa. Appl Anim Husb Rural Dev 2008 1:1–9

[CR24] Smouse RPP, Peakall R (2012) GenAlEx 6.5: genetic analysis in Excel. Population genetic software for teaching and research—an update. Bioinformatics 28:2537–2539. 10.1093/bioinformatics/bts460 22820204 10.1093/bioinformatics/bts460 PMC3463245

[CR23] Statistics South, Africa (2020) Census of Commercial agriculture 2017 Report (11-02-01)

[CR25] Tada O, Muchenje V, Dzama K (2013) Preferential traits for breeding Nguni cattle in low-input in-situ conservation production systems. SpringerPlus 2:195. 10.1186/2193-1801-2-195 23705106 10.1186/2193-1801-2-195 PMC3657091

[CR26] van der Westhuizen L, MacNeil MD, Scholtz MM, Neser FWC, Makgahlela ML, van Wyk JB (2020) Genetic variability and relationships in nine South African cattle breeds using microsatellite markers. Trop Anim Health Prod 52:177–184. 10.1007/s11250-019-02003-z 31388877 10.1007/s11250-019-02003-z

[CR27] Van Marle-Köster E, Visser C, Sealy J, Frantz L (2021) Capitalizing on the potential of South African indigenous beef cattle breeds: a review. Sustainability 13:4388. 10.3390/su1308438

